# Complement component 7 is associated with total- and cardiac death in chest-pain patients with suspected acute coronary syndrome

**DOI:** 10.1186/s12872-021-02306-w

**Published:** 2021-10-14

**Authors:** Reidun Aarsetøy, Thor Ueland, Pål Aukrust, Annika E. Michelsen, Ricardo Leon de la Fuente, Heidi Grundt, Harry Staines, Ottar Nygaard, Dennis W. T. Nilsen

**Affiliations:** 1 Present Address: Department of Clinical Science, University of Bergen, Bergen, Norway; 2 Department of Cardiology, Stavanger University Hospital, P.O. Box 8100, 4068 Stavanger, Norway; 3 Rikshospitalet, Research Institute of Internal Medicine, Oslo University Hospital, Oslo, Norway; 4 Rikshospitalet, Section of Clinical Immunology and Infectious Diseases, Oslo University Hospital, Oslo, Norway; 5 Centro Cardiovascular Salta, Salta, Argentina; 6 Department of Respiratory Medicine, Stavanger University Hospital, Stavanger, Norway; 7 Sigma Statistical Services, Balmullo, UK; 8 Department of Cardiology, Haukeland University Hospital, Bergen, Norway

**Keywords:** Prognostic biomarkers, Acute coronary syndrome, All-cause mortality, Cardiac death, Complement component 7, High-sensitivity C-reactive protein

## Abstract

**Background:**

Complement activation has been associated with atherosclerosis, atherosclerotic plaque destabilization and increased risk of cardiovascular events. Complement component 7 (CC7) binds to the C5bC6 complex which is part of the terminal complement complex (TCC/C5b-9). High-sensitivity C-reactive protein (hsCRP) is a sensitive marker of systemic inflammation and may reflect the increased inflammatory state associated with cardiovascular disease.

**Aim:**

To evaluate the associations between CC7 and total- and cardiac mortality in patients hospitalized with chest-pain of suspected coronary origin, and whether combining CC7 with hsCRP adds prognostic information.

**Methods:**

Baseline levels of CC7 were related to 60-months survival in a prospective, observational study of 982 patients hospitalized with a suspected acute coronary syndrome (ACS) at 9 hospitals in Salta, Argentina. A cox regression model, adjusting for conventional cardiovascular risk factors, was fitted with all-cause mortality, cardiac death and sudden cardiac death (SCD) as the dependent variables. A similar Norwegian population of 871 patients was applied to test the reproducibility of results in relation to total death.

**Results:**

At follow-up, 173 patients (17.7%) in the Argentinean cohort had died, of these 92 (9.4%) were classified as cardiac death and 59 (6.0%) as SCD. In the Norwegian population, a total of 254 patients (30%) died. In multivariable analysis, CC7 was significantly associated with 60-months all-cause mortality [hazard ratio (HR) 1.26 (95% confidence interval (CI), 1.07–1.47) and cardiac death [HR 1.28 (95% CI 1.02–1.60)], but not with SCD. CC7 was only weakly correlated with hsCRP (r = 0.10, *p* = 0.002), and there was no statistically significant interaction between the two biomarkers in relation to outcome. The significant association of CC7 with total death was reproduced in the Norwegian population.

**Conclusions:**

CC7 was significantly associated with all-cause mortality and cardiac death at 60-months follow-up in chest-pain patients with suspected ACS.

***Clinical trial registration*:**

ClinicalTrials.gov Identifier: NCT01377402, NCT00521976.

**Supplementary Information:**

The online version contains supplementary material available at 10.1186/s12872-021-02306-w.

## Introduction

Cardiovascular disease (CVD) is a major contributor to worldwide morbidity and mortality [1]. The search for new prognostic biomarkers to identify patients at increased risk for new cardiovascular events and death is ongoing. In a large-scale proteomic study [2], nine plasma proteins combined were found to predict adverse cardiovascular events in patients with stable coronary heart disease (CHD). One of the proteins selected was complement component 7 (CC7), which is involved in the terminal complement pathway by formation of the terminal complement complex (TCC)/C5b-9 [2, 3].

The complement system is an important component of the innate immune system. However, if the activation is overwhelming or persists too long, it could harm the host and inappropriate complement activation has been shown to play a pathogenic role, not only during acute inflammatory states, but also in chronic inflammatory disorders such as CVD [4, 5]. The complement system is found to play an important role in the induction and progression of atherosclerosis [5, 6]. The presence of complement components, receptors and regulators are demonstrated in the atherosclerotic arterial wall [7, 8], and some reports suggest that complement components, including CC7, may be produced locally within the atherosclerotic plaque, in addition to being retained from the plasma [7, 9]. Complement activation with subsequent TCC/C5b-9 mediated cell-lysis, activation and proliferation of smooth muscle- and endothelial cells, release of chemotactic cytokines and recruitment of inflammatory cells [6], leads to increased severity and complexity of the atherosclerotic lesion, and to plaque destabilization with increased risk of development of an acute coronary syndrome (ACS).

General activation of the complement system is demonstrated in patients with CHD [5, 10], and increased circulating levels of soluble TCC/C5b-9 have been found in patients with an acute myocardial infarction (AMI) [11, 12]. Complement activation has been shown to play a critical role in the pathophysiology of the ischemia–reperfusion injury [5, 13], and may exacerbate the cardiac damage after an AMI [13]. However, to this end, data on CC7 in relation to prognosis following AMI are scarce.

In this context, we hypothesized that increased levels of CC7 may be associated with outcome in patients hospitalized with chest pain of suspected ischemic origin, linking associations to atherosclerotic pathophysiology and risk prediction.

In atherosclerotic lesions of human coronary arteries, a frequent co-localization of C-reactive protein (CRP) and of the TCC/C5b-9 has been observed, suggesting that CRP may be a mediator of atherosclerosis by activation of the complement system [3, 14]. Based on this interaction between CRP and the complement system, we also examined if the combination of these two markers would yield additional prognostic information.

All associations were assessed in an Argentinean population and validated in a similar Norwegian cohort.

## Methods

### Study design and patient population

This study was performed as a part of the ARgentinean Risk Assessment Registry in the Acute Coronary Syndrome (ARRA-RACS) (ClinicalTrials.gov Identifier: NCT01377402), an observational cohort study with the objective to identify early-on prognostic markers in hospital-admitted chest pain patients with clinically suspected ACS. The reproducibility of the association between CC7 and all-cause mortality was assessed in a similar Norwegian cohort, the Risk Markers in the Acute Coronary Syndrome (RACS) (ClinicalTrials.gov Identifier: NCT00521976). The two studies had similar design, and the same protocol and case report form were applied for both cohorts.

A total of 982 patients admitted to nine different hospitals in Salta, Northern Argentina, from December 2005 until January 2009, were recruited in ARRA-RACS, and 871 patients admitted to Stavanger University Hospital, Norway, were consecutively recruited from November 2002 until September 2003 in RACS. Exclusion criteria were age < 18 years, unwillingness or incapacity to provide informed consent and prior inclusion. Troponin T (TnT) levels at baseline and at 6 h after admission were used for subclassification. B-type natriuretic peptide (BNP) was used as a quality indicator of the registry.

In the present study, admission values of CC7 were available for 980 patients, and 978 patients had an available admission value of high-sensitivity (hs) CRP in ARRA-RACS. In RACS, admission levels of CC7 and hsCRP were available in 847 and 868 patients, respectively. Median follow-up time was 42.9 months in ARRA-RACS and 60-months in RACS. The primary outcome was all-cause mortality from time of inclusion until 60-months follow-up. In the ARRA-RACS study we also assessed cardiac death and sudden cardiac death (SCD) at up to 60-months follow-up as secondary endpoints. The definition of cardiac death included death preceded by a definitive myocardial infarction or by chest pain > 20 min without a given TnT, or a history of ischemic heart disease and no other obvious cause of death [15]. Survival status, time and cause of death, and other clinical follow-up data were obtained from hospital- and public registries and by telephone interview at 30 days and 6, 12 and 24 months, as previously described [15]. If needed, additional information was obtained from general practitioners and nursing homes. Later follow-up information was provided from death-registries.

Baseline laboratory and clinical data, including a history of previous myocardial infarction, angina pectoris, heart failure (HF), diabetes mellitus, smoking status (stratified in categories of current smokers, previous smokers or never-smokers), hypercholesterolemia and arterial hypertension, were based on hospital records and personal interviews [15].

Unstable angina pectoris, non-ST segment elevation myocardial infarction (NSTEMI) and ST-segment elevation myocardial infarction (STEMI) were collectively defined as an ACS. For the diagnosis of an AMI, we applied a cut-off value for TnT of 30 ng/L in the Argentinean population and 50 ng/L in the Norwegian population, as specified for the assay in use. Electrocardiographic ST-segment depression or elevation, T-wave inversion or left bundle-branch block were recorded at admission [15]. TnT release >/≤ 10 ng/L was used for risk stratification, as this value represents the lowest detection-limit of the applied assay.

Written informed consent was obtained from all patients. The ARRA-RACS study was approved by the Ethics Committee at the Board of Medical School of Salta, Argentina, and the RACS study was approved by the Regional Board of Research Ethics and by the Norwegian Health authorities. Both studies were conducted in accordance with the Helsinki declaration of 1971, as revised in 1983.

### Blood sampling procedures and laboratory measurements

Blood was drawn immediately following admission by direct venepuncture of an antecubital vein, applying a minimum of stasis. A second blood sample for measurement of TnT was drawn 6–7 h later. All samples were centrifuged for 15 min at 2000 g at 20 °C. Measurement in serum of TnT, creatinine, glucose and lipids were performed immediately after centrifugation. Aliquots of ethylene diamine tetraacetic acid (EDTA) plasma, citrated plasma and serum were frozen and stored at − 80 °C for later measurements.

#### CC7

CC7 (Cat#MBS2099370) levels were analyzed using antibodies from MYBioSource (SanDiego, CA, USA) in duplicate in a 384-well format using a combination of a SELMA (Jena, Germany) pipetting robot and a BioTek (Winooski, VT, USA) dispenser/washer. Absorption was read at 450 nm with wavelength correction set to 540 nm using an ELISA plate reader (BioTek). Intra- and inter-assay coefficients were < 10%.

#### hsCRP, Troponin T and BNP

hsCRP (Tina-quant® C-reactive protein (latex) high-sensitivity assay, Roche Diagnostics, Germany) was analysed in serum on a Roche automated clinical chemistry analyzer (MODULAR P). The lower limit of detection was 0.03 mg/L and the measuring range 0.1–20.0 mg/L with an extended measuring range with automatic re-run 0.1–300 mg/L. The between-assay CV was 3.45% at 1.19 mg/L and 2.70% at 0.43 mg/L, respectively [14].

TnT was quantified in serum by a cardiac-specific fourth generation (ARRA-RACS) and second generation (RACS) TnT ELISA assay from Roche Diagnostics, using a high-affinity cardiac-specific TnT isoform antibody [15]. The lower limit of detection for the assays was 10 ng/L.

BNP [Microparticle Enzyme Immunoassay Abbott AxSYM® (Abbott Laboratories, Abbott Park, Illinois, USA)] was analyzed in EDTA plasma as recommended by the manufacturer and as previously described [15].

### Statistical analysis

Descriptive statistics are presented as medians with interquartile range (25th–75th percentile) for continuous data and as numbers and percentages for categorical data. Differences in baseline characteristics were assessed by the Kruskal–Wallis test for continuous data and the Chi-squared test for categorical data. The Mann–Whitney U test was used to test for the equality of the median of two samples, comparing biomarker-levels in non-survivors with survivors. Due to a skewed distribution, CC7, hsCRP, BNP and estimated glomerular filtration rate (eGFR) levels were logarithmically transformed to the base-e (log_e_) prior to analysis of continuous values and normalised by dividing by the standard deviation (SD). Pearson’s correlation coefficient was calculated to identify a possible relation between the admission level of the two biomarkers, CC7 and hsCRP. A stepwise multivariable linear regression analysis was performed to determine which baseline variables were associated with the admission levels of CC7.

Patients were divided into quartiles (Q1–4) according to their CC7 concentrations. The Kaplan–Meier product limits were used for plotting the times to event and the log-rank test was used to test for the equality of the survival curves. Cox regression models, applying both quartiles and continuous log_e_-transformed values, were fitted for CC7 with all-cause mortality, cardiac death, and SCD within 60-months as the dependent variables in the ARRA-RACS study and all-cause mortality within 60-months follow-up in the RACS study. In multivariable analysis applying a forced-entry method, we adjusted for traditional cardiovascular risk factors, which included age, gender, a medical history of previous CHD (i.e. angina pectoris, myocardial infarction, coronary artery bypass grafting or percutaneous coronary intervention), a history of HF, diabetes mellitus, hypercholesterolemia (total cholesterol > 6.5 mmol/L), smoking status, use of angiotensin converting enzyme inhibitors (ACEI) or angiotensin receptor blockers (ARB), statins and beta blockers, index diagnosis AMI and laboratory parameters (TnT > 10 ng/L, hsCRP, BNP and eGFR). Hazard ratios (HRs) with 95% confidence intervals (CI) were calculated for each of the higher quartiles as compared to quartile 1. For continuous log_e_-transformed values, we employed HR and 95% CI per SD increase of the biomarker. The hazard ratios presented in the results section are 1-SD on the log scale. Subgroup analyses were performed for patients with or without TnT-release above 10 ng/L at index hospitalization. A Cox regression model was fitted with CC7, hsCRP and CC7*hsCRP as independent variables to test for interaction.

Receiver operated characteristics (ROC) analyses for all-cause mortality and cardiac death at 60-months follow-up in the Argentinean population and for 60-months all-cause mortality in the Norwegian population were created for a prediction model including conventional clinical risk factors with stepwise addition of biomarkers. Differences in area under the curve (AUC) were assessed by applying De Long’s test.

Statistics were performed using the statistical package SPSS version 25 (IBM Corp. Armonk, NY). All tests were 2-sided with a significance level of 5% without multiplicity adjustment.

## Results

### The ARRA-RACS study

#### Study population

Median age at enrolment in the total population was 62 (53–72) years, 59.9% were men. At index hospitalization, 39.6% of patients had a TnT release > 10 ng/L, and 344 patients (35.1%) were classified as having an AMI. Baseline characteristics for the patients, stratified according to CC7 quartiles are summarized in Table 1. Increasing age, a previous history of type II diabetes mellitus, increasing BNP and a TnT value > 10 ng/L at index hospitalization were significantly associated with higher admission levels of CC7 (Table 2). Baseline patient characteristics according to an AMI or not at index hospitalization, are given in Additional file 3: Table S1.

**Table 1:** Baseline characteristics according to CC7 (mg/mL) quartiles (Q) in the Argentinean population (ARRA-RACS)

Characteristics	Total n = 980	Q1 n = 245	Q2 n = 245	Q3 n = 245	Q4 n = 245	*p* value
CC7 (mg/mL)	143.6 (112.4–184.7)	93.4 (78.4–103.3)	127.7 (120.0–136.2)	161.3 (152.5–173.1)	225.3 (202.6–259.4)	< 0.001
Age, years	62.0 (53.0–72.0)	59.0 (50.0–67.0)	59.0 (51.0–71.0)	65.0 (55.0–73.0)	67.0 (57.0–77.0)	< 0.001
Male sex	586 (59.8)	157 (64.1)	142 (58.0)	152 (62.0)	135 (55.1)	0.17
*Risk markers at baseline*						
hsCRP mg/L	3.1 (1.4–8.4)	2.5 (1.1–6.9)	3.1 (1.4–7.8)	3.1 (1.3–8.3)	3.7 (1.8–11.0)	0.004
BNP pg/mL	78 (36–180)	63 (32–126)	61 (32–139)	91 (41–180)	111 (51–345)	< 0.001
eGFR ml/min/1.73m^2^	82 (64—98)	85 (71–99)	83 (69–98)	81 (62–99)	78 (57–96)	0.008
Total cholesterol (mmol/L)	4.7 (4.1–5.5)	4.8 (4.2–5.5)	4.9 (4.1–5.6)	4.7 (4.1–5.5)	4.6 (3.8–5.4)	0.017
Acute myocardial infarction*	344 (35.1)	68 (27.8)	66 (27.1)	93 (38.0)	117 (47.8)	< 0.001
TnT release (> 10 ng/L)	387 (39.5)	75 (30.6)	73 (29.9)	103 (42.0)	136 (55.5)	< 0.001
*Risk factors*						
Smoking						0.024
Current smoking	238 (24.8)	62 (25.9)	79 (32.8)	50 (20.8)	47 (19.7)	
Past smoking	536 (55.8)	135 (56.5)	118 (49.0)	140 (58.1)	143 (59.8)	
Hypertension	632 (64.5)	147 (60.0)	153 (62.5)	162 (66.1)	170 (69.4)	0.14
Diabetes mellitus type I	15 (1.6)	3 (1.2)	4 (1.6)	4 (1.7)	4 (1.7)	0.98
Diabetes mellitus type II	185 (19.1)	31 (12.9)	39 (16.0)	47 (19.6)	68 (28.1)	< 0.001
Total cholesterol > 6.5 mmol/L	72 (7.4)	17 (6.9)	15 (6.2)	19 (7.8)	21 (8.6)	0.75
BMI (kg/m^2^)	27.7 (25.3–30.2)	27.7 (25.4–30.2)	27.7 (25.7–30.4)	27.7 (25.0–30.8)	27.4 (24.9–29.4)	0.30
*History of heart disease*						
Angina pectoris	223 (22.8)	45 (18.4)	55 (22.5)	61 (24.9)	62 (25.3)	0.24
Myocardial infarction	93 (9.5)	24 (9.8)	19 (7.8)	18 (7.4)	32 (13.1)	0.12
Previous CABG	46 (4.8)	13 (5.4)	6 (2.5)	10 (4.2)	17 (7.0)	0.11
Previous PCI	97 (9.9)	24 (9.8)	23 (9.4)	23 (9.4)	27 (11.0)	0.92
Heart failure	165 (16.8)	44 (18.0)	38 (15.5)	40 (16.3)	43 (17.6)	0.88
*Treatment prior to admission*						
ACEI/ARB	407 (41.7)	86 (35.1)	96 (39.3)	109 (44.7)	116 (47.5)	0.026
Beta-blocker	252 (26.1)	66 (27.3)	59 (24.3)	62 (25.8)	65 (27.0)	0.88
Statins	92 (9.5)	20 (8.3)	26 (10.7)	22 (9.2)	24 (10.0)	0.82

Data are presented as median (interquartile range) or numbers (%)

CC7, complement component 7; hs-CRP, high-sensitivity C-reactive protein; BNP, B-type natriuretic peptide; eGFR, estimated glomerular filtration rate; TnT, troponin-T; BMI, body mass index; CABG, coronary artery bypass grafting; PCI, percutaneous coronary intervention; ACEI/ARB, angiotensin converting enzyme inhibitor or angiotensin receptor blocker

*For the diagnosis of an acute myocardial infarction, we applied a cut-off value for TnT of 30 ng/L

**Table 2:** Stepwise multivariable linear regression to determine which baseline variables were associated with admission levels of CC7

	ARRA-RACS (Argentina)		RACS (Norway)	
	CC7		CC7	
	Coefficient (95% CI)	*p* value	Coefficient (95% CI)	*p* value
Constant	10.7 (10.3, 11.0)	< 0.001	12.5 (11.5, 13.6)	< 0.001
BNP	0.14 (0.07, 0.20)	< 0.001	0.11 (0.03, 0.19)	0.007
TnT > 10 ng/L	0.26 (0.13, 0.40)	< 0.001	–	–
hsCRP	–	–	0.10 (0.04, 0.17)	0.002
eGFR	–	–	− 0.075 (− 0.15, − 0.001)	0.048
Age/ 10 years	0.12 (0.08, 0.17)	< 0.001	0.14 (0.08, 0.20)	< 0.001
Heart failure	–	–	0.28 (0.12, 0.44)	0.001
DM Type II	0.28 (0.12, 0.43)	0.001	–	–
Use of ACEI/ARB	–	–	0.14 (0.002, 0.28)	0.047

CC7, continuous log_e_-transformed values of complement component 7 divided by its standard deviation; 95% CI, 95% confidence interval; BNP, continuous log_e_ transformed values of B-type natriuretic peptide divided by its standard deviation; hsCRP, continuous log_e_-transformed values of high-sensitivity C-reactive protein divided by its standard deviation; eGFR, estimated glomerular filtration rate divided by its standard deviation; TnT, Troponin-T; DM, diabetes mellitus, ACEI; Angiotensin converting enzyme inhibitor, ARB; Angiotensin receptor blocker

At 60-months follow-up, 173 patients (17.7%) had died, of these were 92 (9.4%) classified as cardiac death and 59 (6.0%) as SCD.

#### CC7 and outcome at up to 60 months follow-up

**Fig. 1:**
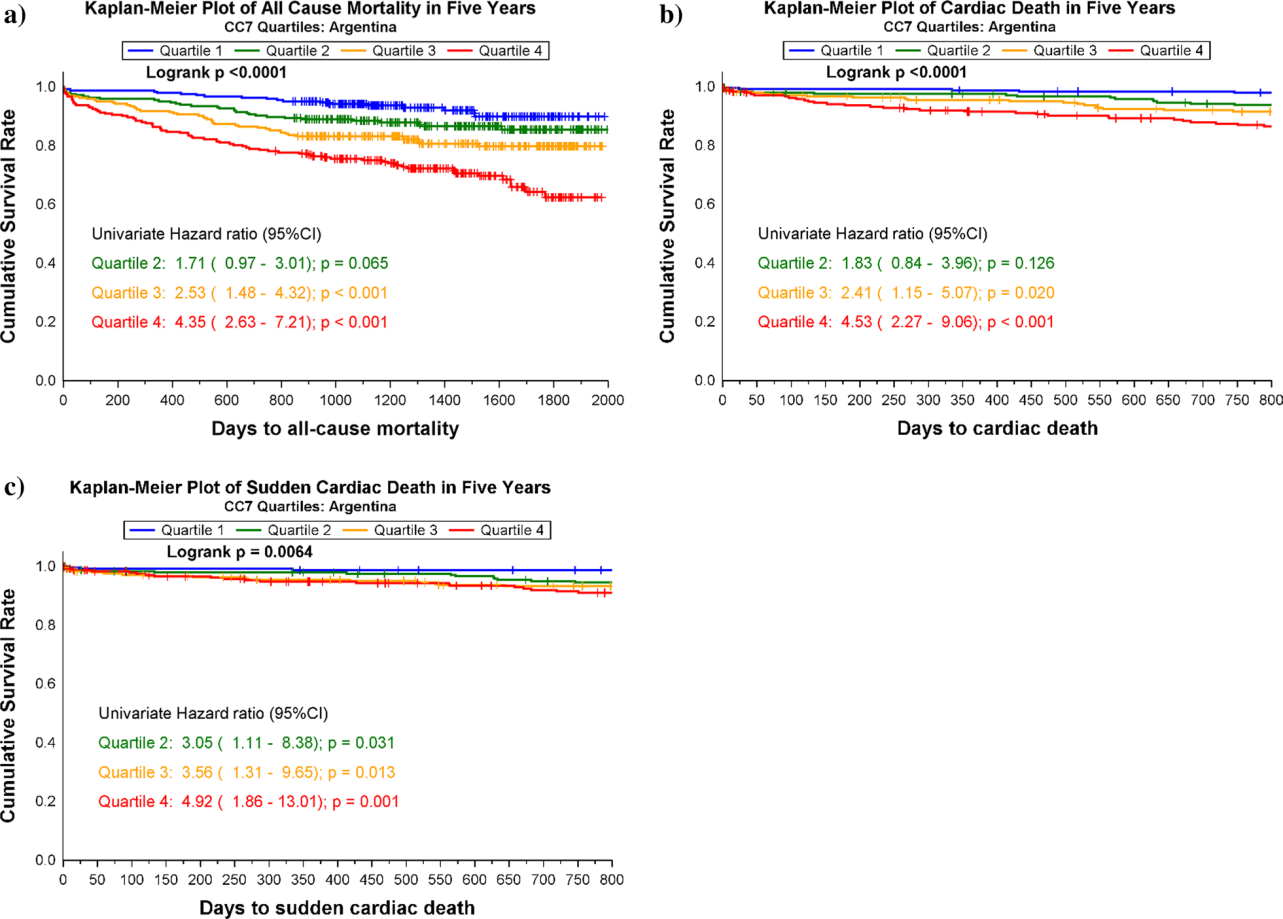
Survival curves by CC7 quartiles for 60-months follow-up in the Argentinean population. **a** all-cause mortality, **b** cardiac death and **c** SCD

**Fig. 2:**
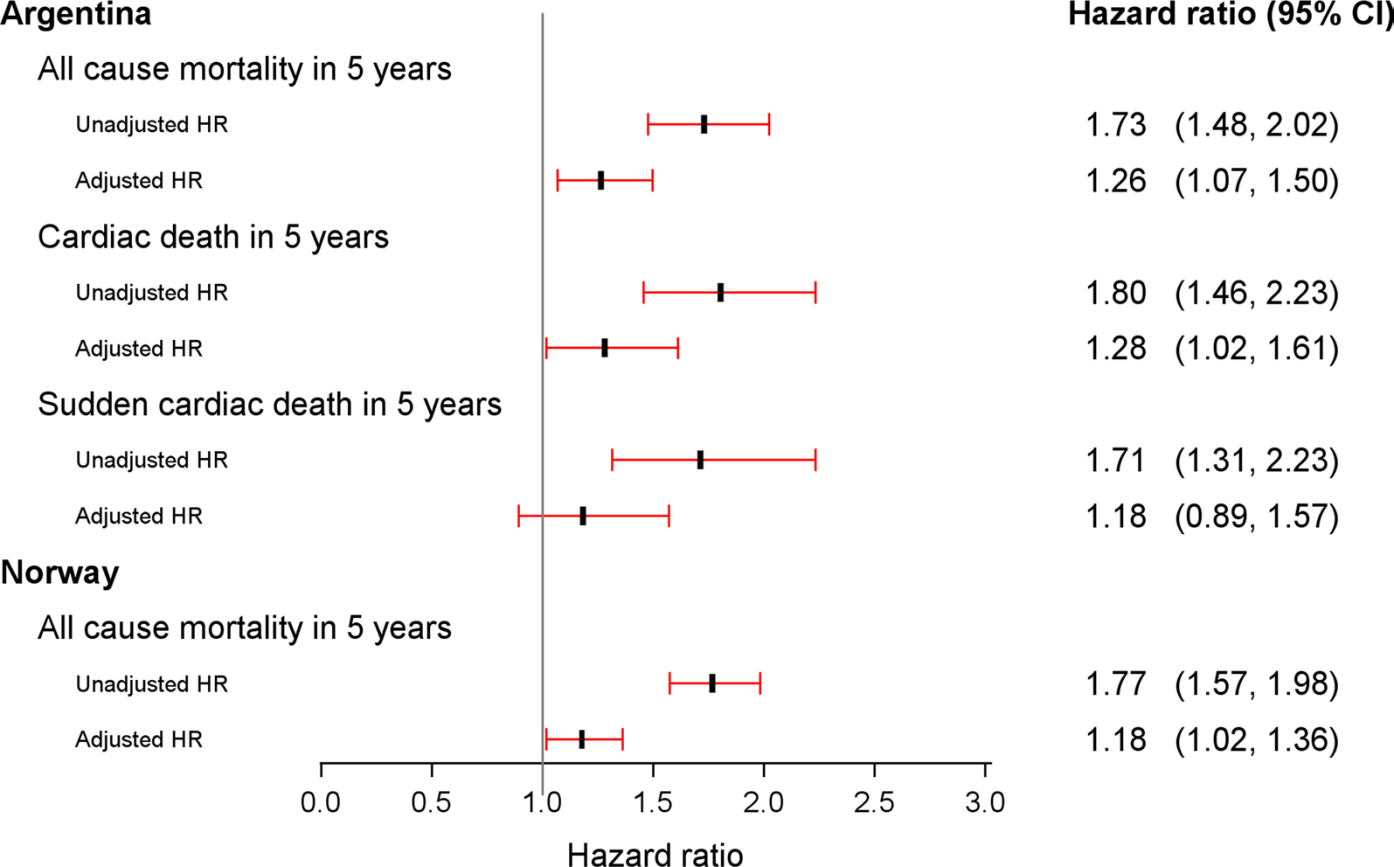
Forest plot of the HR from univariate- and multivariable analysis for CC7 in the Argentinean and Norwegian population, respectively. In multivariable analysis, we adjusted for age, gender, a medical history of previous coronary heart disease (i.e. angina pectoris, myocardial infarction, coronary artery bypass grafting or percutaneous coronary intervention), a history of heart failure, diabetes mellitus, hypercholesterolemia (total cholesterol > 6.5 mmol/L), smoking status, use of angiotensin-converting-enzyme inhibitors or angiotensin receptor blockers, statins and beta blockers, index diagnosis acute myocardial infarction and laboratory parameters (high-sensitivity C-reactive protein (hsCRP), Troponin T, estimated glomerular filtration rate (eGFR) and B-type natriuretic peptide (BNP). CC7, log_e_-transformed value of complement component 7; HR, Hazard Ratio; 95% CI, 95% confidence interval, Unadjusted; univariate analysis, Adjusted; multivariable analysis

CC7 levels were significantly higher in patients who died compared to survivors [median 173.6, 25th–75th percentile: (136.7–225.5) mg/mL vs 138.7 (109.3–176.7) mg/mL, p < 0.001]. In the Kaplan–Meier analysis, increasing quartiles of CC7 were associated with all-cause mortality (p < 0.001), cardiac death (p < 0.001) and SCD (*p* = 0.006), respectively (Fig. 1). Assessed as a continuous variable, CC7 values were significantly associated with all-cause mortality (HR 1.73, 95% CI 1.48–2.02), cardiac death (HR 1.80, 95% CI 1.46–2.23) and SCD (HR 1.71, 1.31–2.23) in the univariate analysis (Fig. 2). In the multivariable analysis, adjusting for established cardiovascular risk factors and prognostic biomarkers, CC7 was independently associated with all-cause mortality (HR 1.26, 95% CI 1.07–1.47) and cardiac death (HR 1.28, 95% CI 1.02–1.60), but was no longer significantly associated with SCD (HR 1.18, 95% CI 0.89–1.57) (Fig. 2).

In unadjusted subgroup analysis of patients stratified according to TnT-release at index hospitalization, there was a significant association between CC7-levels and all-cause mortality in both TnT-positive (HR 1.50, 95% CI 1.23–1.83) and TnT-negative patients (HR 1.64, 95% CI 1.26–2.14). In the multivariable analysis these associations were no longer statistically significant, however a non-significant positive trend remained in the group of patients with a TnT value > 10 ng/L (HR 1.22, 95% CI 1.00–1.50), but not for the TnT-negative patients (HR 1.22, 95% CI 0.92–1.63).

We found a weak, but statistically significant positive correlation between admission levels of CC7 and hsCRP (r = 0.10, *p* = 0.002). There was no statistically significant interaction between CC7 and hsCRP for the prediction of all-cause mortality (HR 0.89, 95% CI 0.77–1.03, for the interaction term), cardiac death (HR 0.96, 95% CI 0.78–1.18) or sudden cardiac death (HR 0.99, 95% CI 0.77–1.27). In multivariable Cox regression analysis, CC7 independently predicted all-cause mortality in line with admission levels of CRP (HR 1.29, 95% CI 1.10–1.51) and BNP (HR 1.47, 95% CI 1.23–1.76), and predicted cardiac death in line with BNP (HR 1.81, 95% CI 1.40–2.36) (Fig. 4a).

The area under the ROC curve for all-cause mortality and cardiac death at 60-months follow-up, respectively, was significantly increased when adding BNP to a risk prediction model including conventional clinical risk factors, TnT and hsCRP (all-cause mortality: *p* = 0.034, cardiac death: *p* = 0.002). Adding CC7 to the risk model of clinical factors and established cardiovascular biomarkers, non-significantly increased the AUC for all-cause mortality at 60-months (*p* = 0.072), Additional file 1: Figure S1.

### The RACS study

#### Study population

Baseline characteristics for the patients, stratified according to CC7 quartiles are summarized in Table 3. The Norwegian population was significantly older than the Argentinean cohort, median age at enrolment was 72.6 (59.0–81.1) years, and 61.3% were men. Compared to the Argentinean cohort, a larger proportion of patients (53.8%) had a TnT-value > 10 ng/L, and 366 patients (43.2%) were classified as having an AMI at index hospitalization. Increasing age, a previous history of HF and use of ACEI or ARB, increasing levels of BNP, CRP and decreasing eGFR were significantly associated with high admission-levels of CC7 (Table 2). Baseline patient characteristics according to an AMI or not at index hospitalization, are given in Additional file 3: Table S2. At 60-months follow-up, 254 patients (30.0%) had died.

**Table 3:** Baseline characteristics according to CC7 (mg/mL) quartiles (Q) in the Norwegian population (RACS)

Characteristics	Total n = 847	Q1 n = 211	Q2 n = 212	Q3 n = 213	Q4 n = 211	*p* value
CC7 (mg/mL)	145.2 (114.0–187.7)	99.5 (87.7–106.3)	129.1 (123.6–138.5)	163.8 (152.7–174.8)	221.2 (203.4–270.9)	< 0.001
Age, years	72.6 (59.0–81.1)	63.1 (50.1–73.5)	69.8 (57.4–79.1)	73.7 (61.7–81.0)	79.5 (71.6–85.5)	< 0.001
Male sex	519 (61.3)	140 (66.4)	129 (60.9)	128 (60.1)	122 (57.8)	0.32
*Risk markers at baseline*						
hsCRP mg/L	4.0 (1.7–13.5)	2.6 (1.2–5.8)	3.5 (1.7–11.3)	4.0 (1.7–14.5)	8.1 (3.0–20.0)	< 0.001
BNP pg/mL	98 (34–310)	49 (16–164)	79 (29–189)	99 (37–334)	247 (94.0–605.0)	< 0.001
eGFR ml/min/1.73m^2^	63 (49–75)	70 (57–81)	65 (55–76)	63 (48–77)	53 (38–67)	< 0.001
Total cholesterol (mmol/L)	5.2 (4.3–6.0)	5.3 (4.4–6.2)	5.4 (4.5–6.1)	5.1 (4.2–5.9)	4.8 (4.1–5.8)	0.009
Acute myocardial infarction*	366 (43.2)	79 (37.4)	104 (49.1)	94 (44.1)	89 (42.2)	0.11
TnT release (> 10 ng/L)	456 (53.8)	91 (43.1)	117 (55.2)	114 (53.5)	134 (63.5)	< 0.001
*Risk factors*						
Smoking						< 0.001
Current smoking	219 (25.9)	82 (38.9)	55 (25.9)	49 (23.0)	33 (15.6)	
Past smoking	311 (36.7)	68 (32.2)	79 (37.3)	77 (36.2)	87 (41.2)	
Hypertension	356 (42.0)	60 (28.4)	93 (43.9)	99 (46.5)	104 (49.3)	< 0.001
Diabetes mellitus type I	8 (0.94)	1 (0.47)	3 (1.4)	3 (1.4)	1 (0.47)	0.57
Diabetes mellitus type II	108 (12.8)	16 (7.6)	24 (11.3)	25 (11.7)	43 (20.4)	< 0.001
Total cholesterol > 6.5 mmol/L	131 (15.5)	38 (18.0)	30 (14.2)	35 (16.4)	28 (13.3)	0.52
BMI (kg/m^2^)	25.3 (22.9–28.0)	25.7 (23.6–28.4)	25.5 (23.2–27.8)	25.0 (22.8–27.5)	24.9 (21.8–27.8)	0.057
*History of heart disease*						
Angina pectoris	374 (44.2)	77 (36.5)	88 (41.5)	96 (45.1)	113 (53.5)	0.004
Myocardial infarction	280 (33.1)	49 (23.2)	70 (33.0)	69 (32.4)	92 (43.6)	< 0.001
Previous CABG	87 (10.3)	20 (9.5)	18 (8.5)	25 (11.7)	24 (11.4)	0.65
Previous PCI	87 (10.3)	22 (10.4)	20 (9.4)	25 (11.7)	20 (9.5)	0.85
Heart failure	227 (26.8)	26 (12.3)	39 (18.4)	53 (24.9)	109 (51.7)	< 0.001
*Treatment prior to admission*						
ACEI/ARB	288 (34.0)	44 (20.9)	60 (28.3)	79 (37.1)	105 (49.8)	< 0.001
Beta-blocker	304 (35.9)	61 (28.9)	80 (37.7)	74 (34.7)	89 (42.2)	0.036
Statins	293 (34.6)	66 (31.3)	65 (30.7)	85 (39.9)	77 (36.5)	0.14

Data are presented as median (interquartile range) or numbers (%). * For the diagnosis of an acute myocardial infarction, we applied a cut-off value for TnT of 50 ng/L

CC7, complement component 7; hs-CRP, high-sensitivity C-reactive protein; BNP, B-type natriuretic peptide; eGFR, estimated glomerular filtration rate; TnT, troponin-T; BMI, body mass index; CABG, coronary artery bypass grafting; PCI, percutaneous coronary intervention; ACEI/ARB, angiotensin converting enzyme inhibitor or angiotensin receptor blocker

#### CC7 and outcome at up to 60 months follow-up

**Fig. 3:**
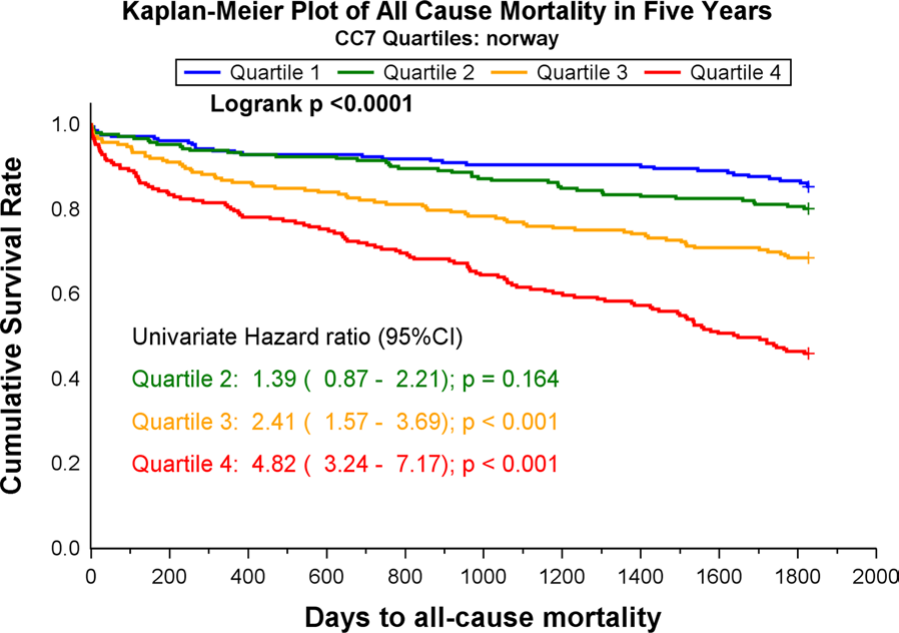
Survival curves by CC7 quartiles for 60-months all-cause mortality in the Norwegian population

CC7 levels were significantly higher in patients who died compared to survivors [median 177.6, 25th–75th percentile: (141.5–219.5) mg/mL vs 134.8 (108.7–167.1) mg/mL, p < 0.001]. In the Kaplan–Meier analysis, increasing quartiles of CC7 were associated with all-cause mortality (p < 0.001) (Fig. 3). Assessed as a continuous variable, CC7 values were significantly associated with all-cause mortality in both the univariate (HR 1.77, 95% CI 1.57–1.98) and the multivariable analysis (HR 1.18, 95% CI 1.02–1.36) (Fig. 2).

In unadjusted subgroup analysis of patients stratified according to TnT-release at index hospitalization, there was a significant association between CC7-levels and all-cause mortality in both TnT-positive- (HR 1.62, 95% CI 1.40–1.87) and TnT-negative patients (HR 1.85, 95% CI 1.53–2.24). In the multivariable analysis, CC7 was independently associated with outcome in TnT-negative patients (HR 1.52, 95% CI 1.16–2.01), but not in TnT-positive patients (HR 1.06, 95% CI 0.88–1.27).

**Fig. 4:**
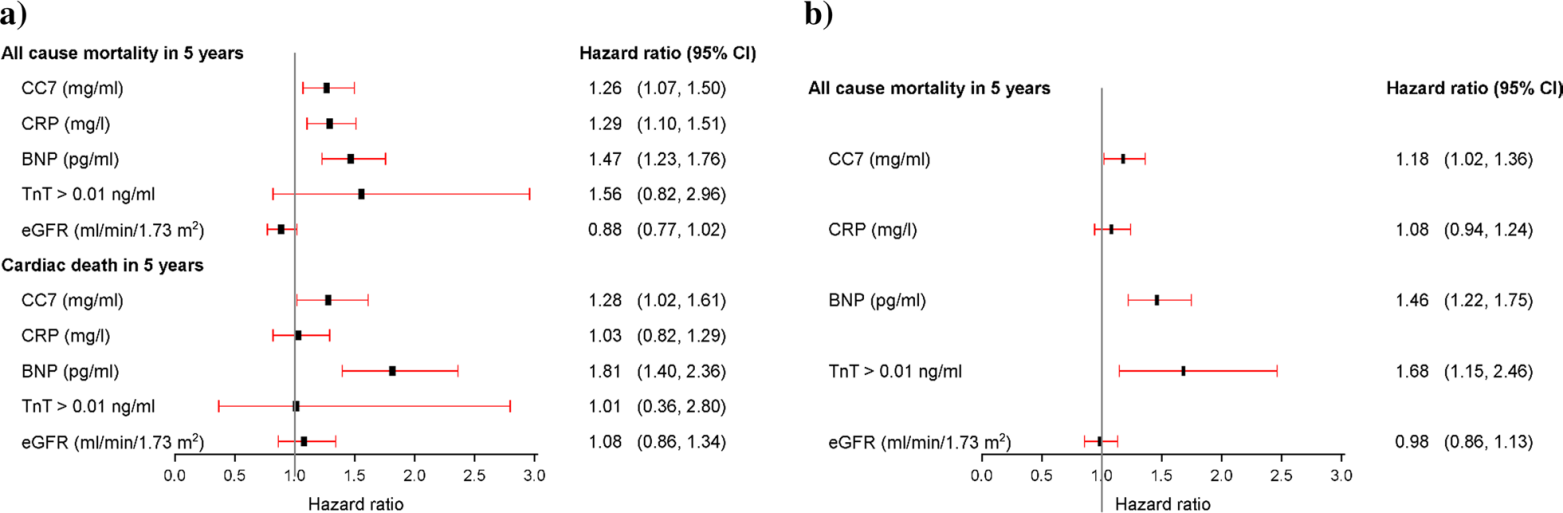
Forest plot of the multivariable analysis for CC7, hsCRP, BNP, TnT and eGFR in the **a** Argentinean population and the **b** Norwegian population. CC7; log_e_-transformed value of complement component 7 divided by its standard deviation, CRP; log_e_-transformed value of high-sensitivity C-reactive protein divided by its standard deviation, BNP; log_e_-transformed value of B-type natriuretic peptide divided by its standard deviation, TnT; Troponin T, eGFR, log_e_-transformed value of estimated glomerular filtration rate divided by its standard deviation

There was a weak, but statistically significant positive correlation between admission levels of CC7 and hsCRP (r = 0.21, p < 0.001). Furthermore, there was a statistically significant interaction between CC7 and hsCRP for the prediction of all-cause mortality (HR 0.82, 95% CI 0.73–0.93, for the interaction term). In multivariable Cox regression analysis, CC7 predicted all-cause mortality in line with BNP (HR 1.46, 95% CI 1.22–1.75) and a TnT value > 10 ng/L (HR 1.68, 95% CI 1.15–2.46) (Fig. 4b).

The area under the ROC curve for all-cause mortality at 60-months follow-up significantly increased with the addition of BNP to the risk model including clinical factors, TnT and hsCRP, *p* = 0.016. Adding CC7 to the risk model resulted in a slight, non-significant increase in the AUC for all-cause mortality, *p* = 0.12 (Additional file 2: Figure S2).

## Discussion

In this prospective observational study of hospital-admitted patients with chest pain of suspected ischemic origin, CC7 was found to be significantly associated with all-cause mortality and cardiac death at 60-months follow-up in the Argentinean population, and the statistically significant association of CC7 with total death was reproduced in the Norwegian population. These associations were present after adjusting for conventional risk factors and established prognostic markers, including BNP, TnT and hsCRP, and may suggest that CC7 could yield additional information regarding outcome in ACS patients.

The complement system is an essential mediator of chronic inflammation, which plays an important role in atherosclerosis. Oxidized- or enzymatically modified low density lipoprotein (LDL), cholesterol crystals, free fatty acids and apoptotic cells inside the atherosclerotic plaque may activate the complement system with subsequent plaque destabilization and increased risk of plaque rupture and development of an ACS [5, 6]. Complement activation may also be the cause of myocardial damage related to ischemia/reperfusion injury following an AMI [5, 10]. We chose to study CC7 as a marker of complement activation and its association to outcome following an ACS, as this complement component previously had been identified as a prognostic marker in stable CHD-patients [2]. In accordance with previous studies [16–21], we found that activation of the common complement pathway was significantly associated with all-cause mortality and cardiac death, respectively. Lindberg et.al. [16] demonstrated that increased levels of the soluble TCC/C5b-9 independently predicted 12-months all-cause mortality and major adverse cardiovascular events (MACE), including cardiovascular mortality, in STEMI-patients. Mellbin et al. [17] found that high admission levels of soluble C5b-9 predicted future cardiovascular events, driven by cardiovascular death, in patients with type 2 diabetes and MI. An increased complement C3/C4 ratio has also been found to be a risk factor for new cardiovascular events in ACS-patients [18]. Moreover, elevated levels of complement C3 were associated with increased risk of new cardiovascular events in women with severe CHD [19], and acted as a predictor of future MI in men without previous manifestation of atherosclerotic disease [20]. Furthermore, Complement C5a was significantly associated with an increased risk of MACE, including death, in patients with pre-existent atherosclerotic disease [21].

The present study is, to the best of our knowledge, the first to report a significant association between CC7 and mortality in intermediate high-risk patients admitted with suspected ACS, a patient population commonly dealt with in the emergency department. Moreover, the association between CC7 and total mortality and cardiac death, respectively, was independent of BNP and TnT, biomarkers that have been found to be strong predictors of outcome in ACS-patients [22, 23]. In multivariable analysis, CC7 was found to predict all-cause mortality and cardiac death in line with BNP, however in a more modest way. In the Norwegian cohort, admission levels of CC7 were found to independently predict outcome, similar to that of TnT-values > 10 ng/L during the first 6 h following hospital admission. When performing subgroup analysis according to TnT release or not, the association of CC7 with total death was attenuated and no longer statistically significant in the Argentinean population, whereas in the Norwegian population, CC7 remained an independent predictor of outcome in TnT-negative patients. As the Norwegian cohort was older than the Argentinean, the atherosclerotic burden would be more extensive in the former cohort, emphasizing the prognostic importance of CC7 in patients with progressive atherosclerosis.

CRP is an acute phase protein and a sensitive and dynamic marker of systemic inflammation. Circulating levels of CRP have been suggested to reflect the extent and severity of general atherosclerosis [14, 24], and to relate to the risk of plaque rupture and vascular thrombosis [24]. Furthermore, an AMI with myocardial necrosis will trigger an acute phase response with a subsequent rise in circulating CRP-levels [14], and elevated levels of CRP have also been reported in patients with both stable- and unstable angina pectoris [25, 26]. Accordingly, hsCRP has been suggested to be a useful marker of cardiovascular risk in both the primary and secondary prevention setting [27]. However, in addition to conventional cardiovascular risk factors and traditional biomarkers, the impact of hsCRP on risk prediction is modest [27–29]. We found that CC7 predicted all-cause mortality in line with hsCRP in the Argentinean population, but there was no significant association between hsCRP and outcome in the multivariable analysis in the Norwegian population. By combining CC7 and hsCRP as independent variables in a Cox regression model, we found a significant positive interaction between the two biomarkers for prediction of all-cause mortality in the Norwegian population, while there was no such interaction in the Argentinean population. Some degree of interaction might exist in an older population due to an age-dependent difference in specificity of hsCRP with respect to atherosclerosis. CC7 was consistently associated with outcome in both populations, whereas hsCRP may be less predictive of CVD in an older population.

Whether increased levels of CRP only reflect the underlying inflammatory state associated with atherosclerosis and heart disease or is directly involved in the pathogenesis of cardiovascular disease, is not clear [14]. CRP co-localizes with complement components in atherosclerotic plaques [6, 14], and in infarcted myocardium [14]. Furthermore, ligand-bound CRP can activate the classical pathway of the complement system [6] and enhance inflammation. Accordingly, we found a weak positive correlation between admission levels of hsCRP and CC7, which is a component of the TCC.

In summary, CC7 binds to C5b-6, anchors the C5b-7 complex to the cell membrane and allows subsequent binding of C8, polymerisation of C9 and finally assembly of the TCC/C5b-9 [3]. Elevated levels of CC7 most likely reflect an inflammatory state with increased formation of the TCC, which is known to be an important mediator of atherosclerosis progression and thromboembolic complications [5, 6]. The association between CC7 and mortality, remained significant even after adjusting for hsCRP-levels, which may be due to CC7 being a more specific marker of CVD.

In the clinical setting, BNP seems to be the strongest predictor of outcome in our patient population. However, adding CC7 to the risk model of conventional clinical risk factors, including TnT, hsCRP and BNP, resulted in a non-significant increase in the predictive accuracy for all-cause mortality in the Argentinean population, with a similar trend in the Norwegian population. Thus, CC7 could play a role in risk prediction of patients with clinically suspected ACS, as part of a multi-marker strategy including different pathophysiological aspects of CHD. This would have to be evaluated in further studies.

## Strengths

Our study had a prospective and observational design. The study population was unaffected by patient selection and interventional regimens prior to blood collection. In our multivariable analyses, we also included prognostic biomarkers in addition to clinical risk factors, to assess the clinical significance of CC7 and hsCRP. Furthermore, as previously [30], we have tested the reproducibility of results in a comparative study population.

## Limitations

CC7 and hsCRP are based on one blood sample harvested at hospital admission. Furthermore, they may not reflect a steady-state situation, as the levels of biomarkers may have been influenced of the time from symptom onset to hospital admission. We did not adjust for left ventricle ejection fraction (LVEF), but did adjust for known HF and BNP. In the Norwegian population we had only all-cause mortality as an outcome variable at 60-months follow up. However, as previously described [31], at 24-months follow-up, only 9.4% of total deaths were due to cancer. This early recording may reflect the rate of cancer mortality during the long-term follow-up, suggesting that deaths at 60-months follow-up are essentially of cardiac origin.

## Conclusions

CC7 was significantly associated with all-cause mortality and cardiac death at 60-months follow-up in chest-pain patients with suspected ACS. Its clinical relevance is still unclear.

## Supplementary Information

**Additional file 1: Figure S1**. Receiver operated characteristic curve for a prediction model including conventional clinical risk factors with the addition of established cardiovascular biomarkers (TnT, hsCPR and BNP) and CC7 for the evaluation of a) 60-months all-cause mortality and b) 60-months cardiac death in the Argentinean population.

**Additional file 2: Figure S2**. Receiver operated characteristic curve for a prediction model including conventional clinical risk factors with the addition of established cardiovascular biomarkers (TnT, hsCPR and BNP) and CC7 for the evaluation of 60-months all-cause mortality in the Norwegian population.

**Additional file 3: Table S1**. Baseline characteristics of the Argentinean population (ARRA-RACS) stratified according to an AMI or not at index hospitalization. **Table S2** Baseline characteristics of the Norwegian population (RACS) stratified according to an AMI or not at index hospitalization.

## Data Availability

Local database. The database is administered by the Research Group under the leadership of Prof. Dennis W.T. Nilsen, as described in ClinicalTrials.gov (NCT01377402, NCT00521976). The datasets analysed during the current study are not publicly available, but are available from the corresponding author on reasonable request.

## Abbreviations

ACEI: Angiotensin converting enzyme inhibitor
ACS: Acute coronary syndrome
AMI: Acute myocardial infarction
ARB: Angiotensin receptor blocker
ARRA-RACS: ARgentinean Risk Assessment Registry in the Acute Coronary Syndrome
BNP: B-type natriuretic peptide
CC7: Complement component 7
CHD: Coronary heart disease
CI: Confidence interval
CRP: C-reactive protein
CVD: Cardiovascular disease
EDTA: Ethylene diamine tetraacetic acid
eGFR: Estimated glomerular filtration rate
HF: Heart failure
HR: Hazard ratio
hs: High sensitivity
LDL: Low density lipoprotein
MACE: Major adverse cardiovascular events
NSTEMI: Non-ST segment elevation myocardial infarction
RACS: Risk Markers in the Acute Coronary Syndrome
SCD: Sudden cardiac death
SD: Standard deviation
STEMI: ST-segment elevation myocardial infarction
TCC: Terminal complement complex
TnT: Troponin T
