# Supplementary figures and images for: Complement component 7 is associated with total- and cardiac death in chest-pain patients with suspected acute coronary syndrome
Source: BMC Cardiovasc Disord. 2021 Oct 14;21:496. doi: 10.1186/s12872-021-02306-w (PMC8515738; doi:10.1186/s12872-021-02306-w)

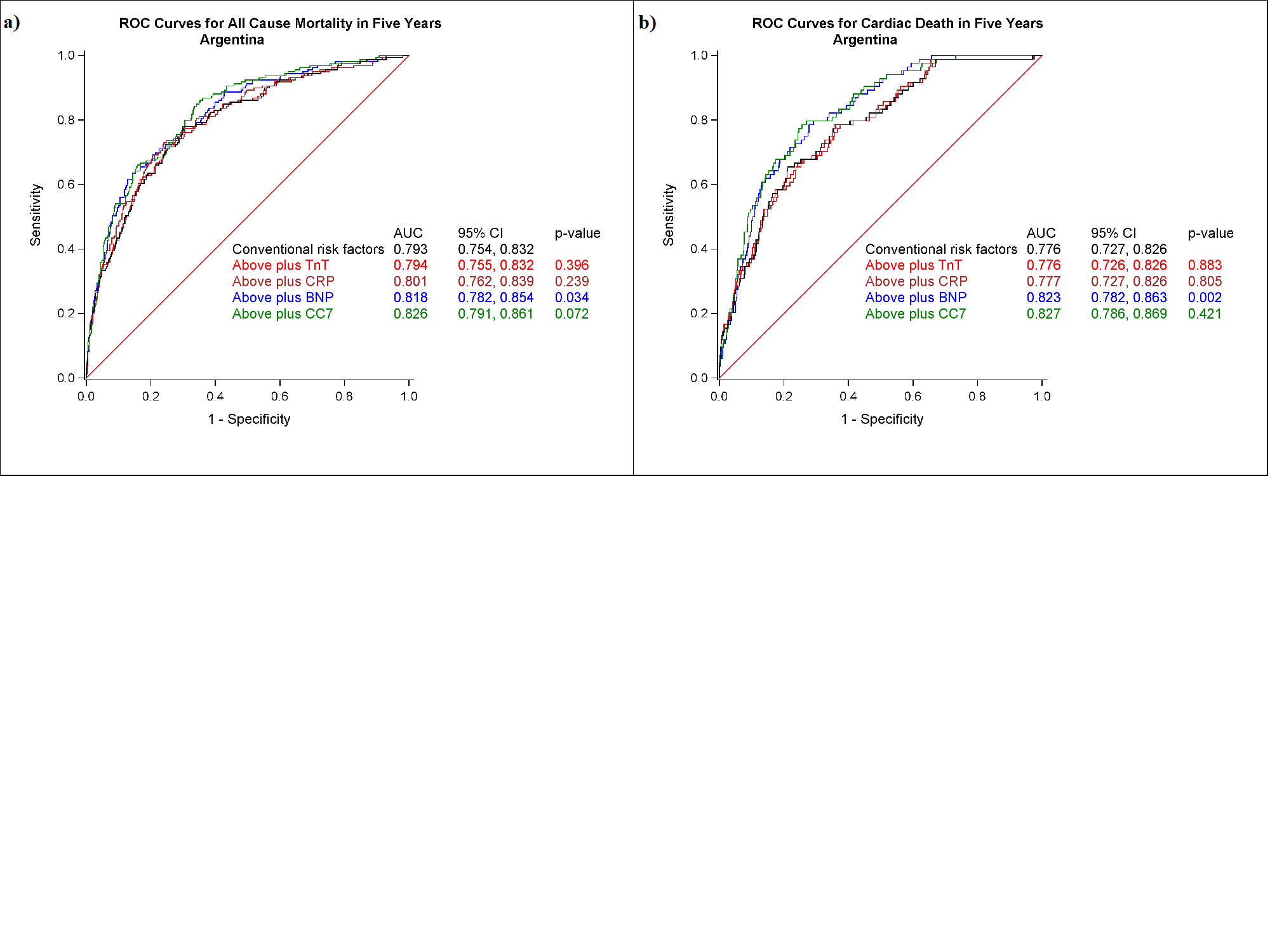

Supplement: Supplementary file 1 — Additional file 1: Figure S1. Receiver operated characteristic curve for a prediction model including conventional clinical risk factors with the addition of established cardiovascular biomarkers (TnT, hsCPR and BNP) and CC7 for the evaluation of a) 60-months all-cause mortality and b) 60-months cardiac death in the Argentinean population. [file 12872_2021_2306_MOESM1_ESM.tif]

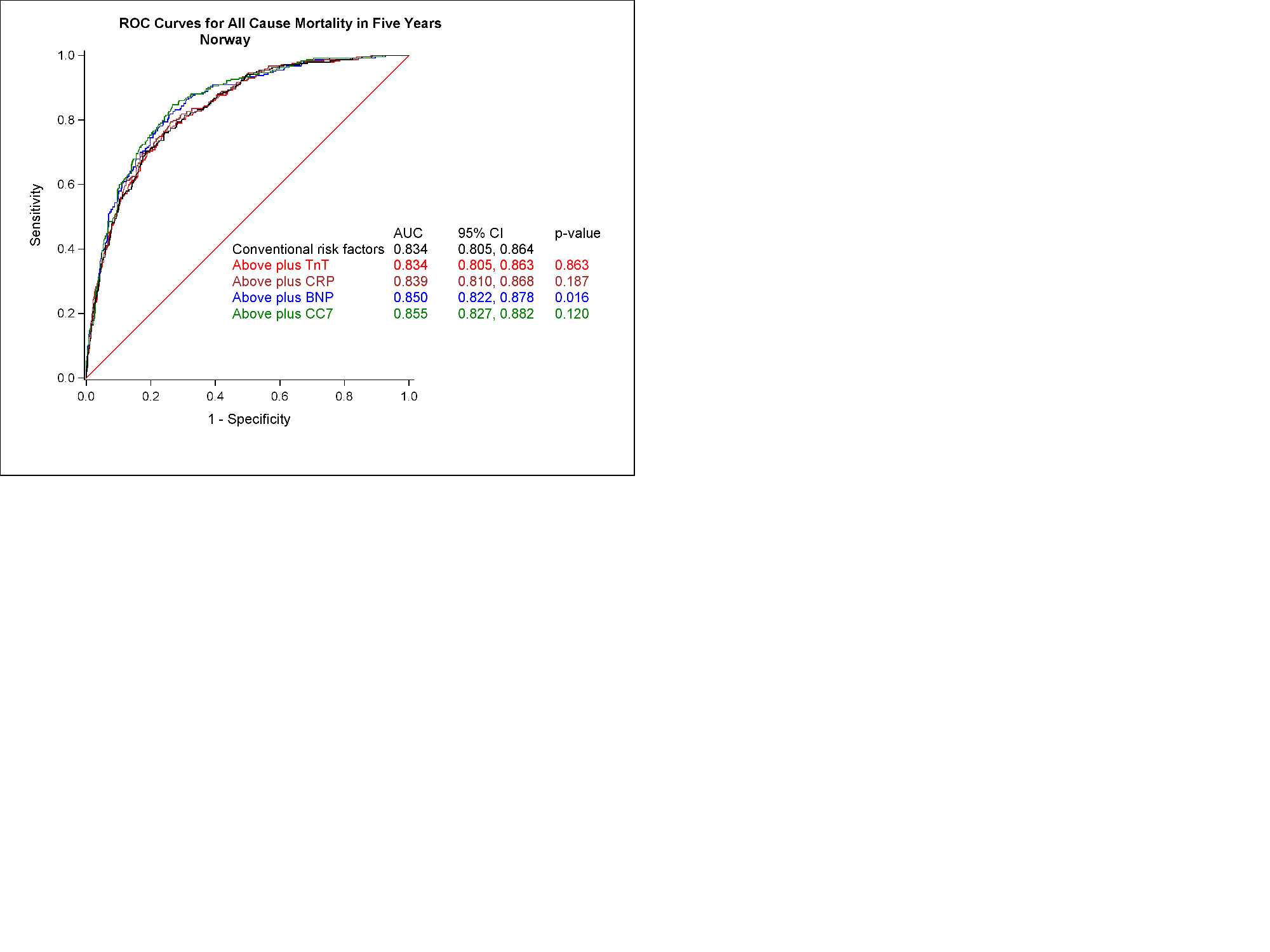

Supplement: Supplementary file 2 — Additional file 2: Figure S2. Receiver operated characteristic curve for a prediction model including conventional clinical risk factors with the addition of established cardiovascular biomarkers (TnT, hsCPR and BNP) and CC7 for the evaluation of 60-months all-cause mortality in the Norwegian population. [file 12872_2021_2306_MOESM2_ESM.tif]
