# Supplementary material for: Complement component 7 is associated with total- and cardiac death in chest-pain patients with suspected acute coronary syndrome
Source: BMC Cardiovasc Disord. 2021 Oct 14;21:496. doi: 10.1186/s12872-021-02306-w (PMC8515738; doi:10.1186/s12872-021-02306-w)
Supplement: Supplementary file 3 — Additional file 3: Table S1. Baseline characteristics of the Argentinean population (ARRA-RACS) stratified according to an AMI or not at index hospitalization. Table S2 Baseline characteristics of the Norwegian population (RACS) stratified according to an AMI or not at index hospitalization. [file 12872_2021_2306_MOESM3_ESM.docx]

**Supplemental Table 1.** **Baseline characteristics of the Argentinean population stratified according to an AMI or not at index hospitalization.**

| **Characteristics:** | **AMI**  **N = 344** | **No AMI**  **N = 637** | **P-value** |
| --- | --- | --- | --- |
|  |  |  |  |
| Age, years | 65.0 (55.0 – 73.0) | 61.0 (51.0 – 70.0) | <0.001 |
| Male sex | 230 (66.7) | 358 (56.2) | 0.001 |
| **Risk markers at baseline;** |  |  |  |
| hs-CRP mg/L | 4.6 (2.0 – 14.3) | 2.5 (1.1 – 6.4) | <0.001 |
| BNP pg/mL | 137 (58 – 324) | 63 (33 – 122) | <0.001 |
| eGFR ml/min/1.73m^2^ | 78 (57 – 97) | 83 (68 – 99) | <0.001 |
| Total cholesterol (mmol/L) | 4.7 (4.0 – 5.6) | 4.7 (4.1 – 5.5) | 0.84 |
| TnT release (> 0.01 ng/mL) | 344 (100) | 44 (6.9) | <0.001 |
| **Risk factors;** |  |  |  |
| Smoking |  |  | 0.008 |
| Current smoking | 98 (29.0) | 140 (22.5) |  |
| Past smoking | 166 (49.1) | 371 (59.6) |  |
| Hypertension | 213 (61.9) | 420 (65.9) | 0.21 |
| Diabetes mellitus type I | 7 (2.1) | 8 (1.3) | 0.34 |
| Diabetes mellitus type II | 82 (24.2) | 105 (16.7) | 0.005 |
| Total cholesterol > 6.5 mmol/L | 30 (8.8) | 42 (6.6) | 0.21 |
| BMI (kg/m^2^) | 27.5 (24.9 – 30.2) | 27.7 (25.4 – 30.3) | 0.19 |
| **History of heart disease;** |  |  |  |
| Angina pectoris | 84 (24.4) | 139 (21.8) | 0.35 |
| Myocardial infarction | 41 (11.9) | 53 (8.3) | 0.068 |
| Previous CABG | 22 (6.5) | 25 (4.0) | 0.083 |
| Previous PCI | 43 (12.5) | 55 (8.6) | 0.054 |
| Heart failure | 79 (23.0) | 86 (13.5) | <0.001 |
| **Treatment prior to admission;** |  |  |  |
| ACEI/ARBs | 148 (43.4) | 259 (40.7) | 0.41 |
| Beta-blockers | 88 (26.1) | 165 (26.2) | 0.98 |
| Statins | 37 (11.0) | 56 (8.9) | 0.29 |

Data are presented as median (interquartile range) or numbers (%).

For the diagnosis of an acute myocardial infarction, we applied a cut-off value for TnT of 0.03 ng/mL.

Abbreviations: hs-CRP, high-sensitivity C-reactive protein; BNP, B-type natriuretic peptide; eGFR, estimated glomerular filtration rate; TnT, troponin-T; BMI, body mass index; CABG, coronary artery bypass grafting; PCI, percutaneous coronary intervention; ACEI/ARB, angiotensin converting enzyme inhibitor or angiotensin receptor blocker.

**Supplemental Table 2.** **Baseline characteristics of the Norwegian population stratified according to an AMI or not at index hospitalization.**

| **Characteristics:** | **AMI**  **N = 380** | **No AMI**  **N = 491** | **P-value** |
| --- | --- | --- | --- |
|  |  |  |  |
| Age, years | 72.6 (60.4 – 80.8) | 72.7 (58.0 – 81.3) | 0.60 |
| Male sex | 258 (67.9) | 273 (55.6) | <0.001 |
| **Risk markers at baseline;** |  |  |  |
| hs-CRP mg/L | 5.0 (2.3 – 16.0) | 3.3 (1.4 – 11.8) | <0.001 |
| BNP pg/mL | 128 (43 – 408) | 83 (28 – 243) | <0.001 |
| eGFR ml/min/1.73m^2^ | 64 (48 – 77) | 63 (49 – 75) | 0.50 |
| Total cholesterol (mmol/L) | 5.3 (4.5 – 6.1) | 5.0 (4.2 – 5.9) | 0.005 |
| TnT release (> 0.01 ng/mL) | 380 (100) | 91 (18.5) | <0.001 |
| **Risk factors;** |  |  |  |
| Smoking |  |  | <0.001 |
| Current smoking | 128 (33.7) | 101 (20.6) |  |
| Past smoking | 132 (34.7) | 183 (37.3) |  |
| Hypertension | 155 (40.8) | 212 (43.2) | 0.48 |
| Diabetes mellitus type I | 6 (1.6) | 3 (0.6) | 0.16 |
| Diabetes mellitus type II | 50 (13.2) | 62 (12.6) | 0.82 |
| Total cholesterol > 6.5 mmol/L | 61 (16.1) | 77 (15.7) | 0.88 |
| BMI (kg/m^2^) | 25.0 (22.9 – 28.0) | 25.5 (22.9 – 28.0) | 0.59 |
| **History of heart disease;** |  |  |  |
| Angina pectoris | 154 (40.5) | 227 (46.2) | 0.092 |
| Myocardial infarction | 118 (31.1) | 172 (35.0) | 0.22 |
| Previous CABG | 30 (7.9) | 58 (11.8) | 0.057 |
| Previous PCI | 27 (7.1) | 60 (12.2) | 0.013 |
| Heart failure | 96 (25.3) | 139 (28.3) | 0.32 |
| **Treatment prior to admission;** |  |  |  |
| ACEI/ARBs | 120 (31.6) | 175 (35.6) | 0.21 |
| Beta-blockers | 116 (30.5) | 197 (40.1) | 0.003 |
| Statins | 109 (28.7) | 189 (38.5) | 0.002 |

Data are presented as median (interquartile range) or numbers (%).

For the diagnosis of an acute myocardial infarction, we applied a cut-off value for TnT of 0.05 ng/mL.

Abbreviations: hs-CRP, high-sensitivity C-reactive protein; BNP, B-type natriuretic peptide; eGFR, estimated glomerular filtration rate; TnT, troponin-T; BMI, body mass index; CABG, coronary artery bypass grafting; PCI, percutaneous coronary intervention; ACEI/ARB, angiotensin converting enzyme inhibitor or angiotensin receptor blocker.
